# Knowledge, attitude and practice regarding lifestyle modification in type 2 diabetic patients

**DOI:** 10.4102/phcfm.v6i1.655

**Published:** 2014-12-09

**Authors:** Henry I. Okonta, John B. Ikombele, Gboyega A. Ogunbanjo

**Affiliations:** 1 Department of Family Medicine and Primary Health Care, University of Limpopo, Medunsa Campus, Pretoria; 2 Mamelodi District Hospital, Pretoria

## Abstract

**Background:**

The number of persons suffering from type 2 diabetes mellitus continues to rise worldwide and causes significant morbidity and mortality, especially in the developing world. Behaviour change and adoption of healthy lifestyle habits help to prevent or slow down the complications of type 2 diabetes mellitus. However, the knowledge and practice of healthy lifestyles in many diabetic patients have been inadequate.

**Aim:**

This study sought to establish the knowledge, attitude and practice regarding lifestyle modification amongst type 2 diabetic patients.

**Setting:**

The diabetic clinic of Mamelodi hospital, Pretoria, Gauteng Province, South Africa.

**Methods:**

A cross-sectional study was done using a structured questionnaire amongst 217 type 2 diabetic patients seen at the diabetic clinic of Mamelodi hospital. Baseline characteristics of the participants were obtained and their knowledge, attitude and practice regarding lifestyle modification were assessed.

**Results:**

Of the 217 participants, 154 (71%) were obese and 15 (7%) were morbidly obese. The majority of respondents (92.2%) had poor knowledge of the benefits of exercise, weight loss and a healthy diet. What is interesting is that the majority (97.7%) demonstrated bad practices in relation to lifestyle modifications, although over four-fifths (84.3%) had a positive attitude toward healthy lifestyle modifications.

**Conclusion:**

Despite the positive attitudes of respondents toward healthy lifestyle modifications, the knowledge and practice regarding lifestyle modifications amongst type 2 diabetes mellitus participants seen at Mamelodi hospital were generally poor.

## Abstrak

**Connaissances, attitudes et pratiques concernant le changement du mode de vie des diabétiques de type 2**.

**Contexte:**

Le nombre de personnes souffrant du diabète sucré de type 2 continue à augmenter dans le monde entier et est une cause importante de morbidité et de décès, surtout dans les pays en voie de développement. Les changements de comportement et l'adoption d'un mode de vie sain permet de prévenir ou de ralentir les complications du diabète sucré de type 2. Cependant, la connaissance et la pratique d'un mode de vie sain sont insuffisantes chez de nombreux diabétiques.

**Objectif:**

Cette étude a cherché à déterminer les connaissances, attitudes et pratiques relatives à la modification du mode de vie des diabétiques de type 2.

**Lieu:**

La clinique pour diabètes de l'hôpital de Mamelodi, à Pretoria, Province de Gauteng, Afrique du Sud.

**Méthodes:**

On a effectué une étude transversale au moyen d'un questionnaire structuré parmi 217 diabétiques de type 2 examinés à la clinique du diabète de l'hôpital de Mamelodi. On a collecté les caractéristiques de base des participants et évalué leurs connaissances, attitudes et pratiques relatives à la modification de leur mode de vie.

**Résultats:**

Des 217 participants, 154 (71%) étaient obèses et 15 (7%) étaient d'une obésité excessive. La majorité des répondants (92.2%) ne savaient presque rien des bienfaits de l'exercice physique, de la perte de poids et d'un régime alimentaire sain. Il est intéressant de noter que la majorité (97.7%) n'avait pas modifié son mode vie tandis que plus des quatre cinquième d'entre eux (84.3%) avaient une attitude positive envers les modifications relatives à un mode de vie sain.

**Conclusion:**

En dépit des attitudes positives des répondants envers les modifications relatives à un mode vie sain, les participants diabétiques de type 2 examinés à l'hôpital de Mamelodi avaient une mauvaise connaissance et pratique de la modification du mode de vie.

## Introduction

### Social value

The burden of type 2 diabetes mellitus is becoming an epidemic and is a cause of morbidity and mortality, especially in the developing world. Worldwide, the prevalence of type 2 diabetes mellitus was 382 million in 2013 and it is estimated to rise to 592 million by 2035.^1^ The prevalence of people living with type 2 diabetes mellitus in Africa was 20 million in 2013 and is projected to go through a two-fold increase over the next 22 years (i.e., by 2035).^1^ The prevalence of type 2 diabetes mellitus in South Africa is amongst the highest in sub-Saharan Africa, with 2 million people known to have the condition and more than 1.5 million people with undiagnosed diabetes mellitus.^2^ In a South African study in 2006, the greatest prevalence of type 2 diabetes mellitus was found amongst the Indian community of Durban (13%) and the elderly (mixed-race) community of Cape Town (28.7%).^3^ Another study in South Africa confirmed the high prevalence of 28.2% of type 2 diabetes mellitus amongst the South African mixed-race population.^4^ The main driving force for this current worldwide epidemic of type 2 diabetes mellitus is environmental. Urbanisation encourages a sedentary lifestyle, with a lack of participation in physical activities. This can lead to obesity, which is an important factor in developing type 2 diabetes mellitus through insulin resistance.^5^

### Scientific value

Lifestyle modification is vital in the management of type 2 diabetes mellitus. Studies on the knowledge, attitude and practice of lifestyle modification, specifically with regard to type 2 diabetic patients, are very few, with varied results from various settings. In a study of 100 type 2 diabetic patients in Malaysia, 87% of the participants were knowledgeable, with a score of 50% or more on knowledge questions; 98% had a score of 50% or more on attitude questions; and 99% had a score of 50% or more on lifestyle modification practices scores.^6^ However, in another study of 207 type 2 diabetic patients in India, 83.3% of participants had poor knowledge of lifestyle modification as a non-pharmacologic treatment for diabetes mellitus.^7^ Furthermore, in a study of 1982 diabetic patients in Kenya, 28% of the participants had a good attitude toward lifestyle modification; 75% had poor dietary practice; and 72% did not exercise regularly.^8^

### Conceptual framework

We observed that most patients seen at the diabetic clinic of Mamelodi hospital were obese, with suboptimal control of their blood glucose levels. In addition, some also had hypertension, diabetic retinopathy and diabetic feet. Encounters with these patients revealed that most of them had poor dietary and physical exercise habits despite the fact that they had been counseled at least once about lifestyle modification.

### Aim and objectives

Within our context, there was no previous study on this topic in this black-populated setting of Mamelodi Township in South Africa. The aim of this study was to explore the knowledge, attitude and practice regarding lifestyle modifications amongst type 2 diabetic patients seen at Mamelodi hospital, east of Pretoria.

## Research methods and design

### Setting

We conducted this study in the diabetic clinic at Mamelodi hospital, a district hospital east of Pretoria in Gauteng Province, South Africa. At the time of this study there was no organised medical nutrition programme to support diabetic patients at Mamelodi hospital.

### Study design

This was a cross-sectional study. The study population was all diagnosed type 2 diabetes mellitus patients seen at the diabetic clinic of Mamelodi hospital from 05 December 2010 to 05 January 2011.The inclusion criteria were type 2 diabetic patients aged 30 years and above seen at Mamelodi hospital for regular follow-ups and who consented to participate in the study.

### Sample population and sampling structure

The sample size was calculated using Epi Info™ version 3.2 (US Centers for Disease Control and Prevention, Atlanta, GA 2004). With an estimated total of 500 type 2 diabetes mellitus patients who attend the diabetic clinic monthly, an expected prevalence of 50% type 2 diabetes mellitus patients with good knowledge, positive attitude and good practice of lifestyle modification, using a 95% confidence level and a 5% degree of error, the sample size was 217. The patients who met the inclusion criteria were selected through systematic sampling of every second patient until the last patient in the sample was selected.

### Data collection

The data for this study were collected using a structured questionnaire. The questionnaire used in this study was created by using questions from existing validated questionnaires in the ‘spoken knowledge in low literacy in diabetes knowledge assessment scale’ (SKILLD^9^) and similar researches by Ambigapathy et al.^6^ in 2003 and Palaian et al.^10^ in 2006. Knowledge questions were obtained from the SKILLD questionnaire,^9^ whilst attitude and practice questions were obtained from the questionnaires developed by Ambigapathy^6^ and Palaian.^10^ Identifying information, such as names of participants, was omitted from the questionnaires.

The questionnaire was administered by the researcher and two qualified professional nurses trained as research assistants and who were proficient in Sepedi and Isizulu (local languages predominantly spoken in Mamelodi Township). Participants who agreed to participate in the study signed informed consent. The data collected from the participants included baseline demographic characteristics, their average monthly income, anthropometric measurements and information on lifestyle modification knowledge, attitude and practice.

### Scoring on the questionnaires

The lifestyle modification knowledge, attitude and practice questionnaire had 6 questions on knowledge regarding the benefits of exercise and weight loss, 12 questions on knowledge about healthy diet, 4 questions on attitudes toward lifestyle modifications and 3 questions on lifestyle modification practices. Each correct answer by the participant for a question earned a score of ‘1’ and each incorrect answer earned a score of ‘0’.

### Reliability and validity

A pilot study on 10 type 2 diabetes mellitus patients who were not included in the study was done and the questionnaire was refined according to the feedback received from the pilot study.

The Isizulu and Sepedi versions of the questionnaire were tested by translation back to English by a different accredited translator and were found to be comparable.

### Data analysis

The data from the questionnaires were captured in Microsoft^®^ Excel 2007 and exported to SPSS Statistics for Windows, Version 17.0 (SPSS Inc., Chicago 2008) where they were analysed by a statistician using descriptive statistics. Linear correlation and *p*-values were calculated to ascertain the statistical significance of key findings.

The results of the analysis were presented in frequency distribution tables. For the 6 questions on knowledge regarding the benefits of exercise and weight loss, participants who had zero to 2 correct answers out of the 6 were assumed to have poor knowledge about the benefits of exercise and weight loss. This score range of 0–2 was labeled as ‘poor knowledge’ in the frequency distribution table. Participants who had between 3 and 4 correct answers were assumed to have average knowledge about the benefits of exercise and weight loss and this score range of 3–4 was labeled as ‘average knowledge’. Participants with 5 to 6 correct answers were assumed to have good knowledge about the benefits of exercise and weight loss, with this score range of 5–6 being labeled as ‘good knowledge’.

Similarly, for the 12 questions on knowledge regarding a healthy diet, score ranges of 0–5, 6–9 and 10–12 were labeled as poor knowledge, average knowledge and good knowledge, respectively. For the 18 global knowledge questions, score ranges of 0–8, 9–13 and 14–18 were labeled as poor knowledge, average knowledge and good knowledge, respectively. For the 3 practice of lifestyle modification questions, scores of 0, 1, 2 and 3 were labeled as very poor practice, poor practice, good practice and very good practice, respectively.

### Ethical considerations

The Medunsa Research Ethics Committee (MREC) provided ethical clearance for our study (clearance certificate number: MREC/M/224/2010:PG). The management of Mamelodi hospital provided permission to conduct the study. Informed consent was obtained from the participants; both the administration and collection of the questionnaires were anonymous and all information volunteered was kept in strict confidence.

## Results

### Baseline characteristics of the respondents

Two hundred and seventeen type 2 diabetes mellitus patients were recruited for the study. Table 1 shows the age group distribution of the participants with the 51- to 60-year age group, within which the majority of the participants fell (*n* = 93, 42.9%). It also shows that a majority of the participants either had no formal education (*n* = 108, 49.5%) or primary school education (*n* = 95, 43.9%); and they had a very low average monthly income – only 1 (0.5%) earning above R4000 (approximately USD 400) per month. Only 7.4% (*n* = 16) had a normal body mass index (BMI); for the others, some were overweight (*n* = 47, 21.7%) and 70.9% (*n* = 154) were obese, with 15 (6.9%) falling in the class 3 obesity subgroup.

**TABLE 1:** Baseline characteristics of participants.

Baseline characteristic	Number	Percentage
**Age group (years)**		
30–40	16	7.4
41–50	47	21.6
51–60	93	42.9
61–70	46	21.2
>70	15	6.9
**Total**	**217**	**100**
**Level of education**		
No formal education	108	49.5
Primary school	95	43.9
High school	14	6.6
Tertiary education	0aa	0.0
**Total**	**217**	**100**
**Average monthly income (Rands)**		
< 500	40	17.9
500–999	55	24.5
1000–1999	111	52.4
2000–4000	10	4.7
>4000	1	0.5
**Total**	**217**	**100**
**BMI (Kg/m**^2^**)**		
Normal (18.5–24.9)	16	7.4
Overweight (25.0–29.9)	47	21.7
Obese class 1 (30.0–34.9)	94	43.3
Obese class 2 (35.0–39.9)	45	20.7
Obese class 3 (40 or more)	15	6.9
**Total**	**217**	**100**

BMI, body mass index.

### Knowledge about lifestyle modification

Table 2 shows that 92.1% (*n* = 200) of the participants had poor knowledge regarding the benefits of exercise and weight loss; 73.3% (*n* = 159) had poor knowledge regarding a healthy diet and 92.6% (*n* = 201) had poor global knowledge regarding the value of exercise, weight loss and a healthy diet.

**TABLE 2:** Frequency distribution of knowledge about lifestyle modification, diet and global knowledge scores.

Variable	Number	Percentage
**Score on knowledge about benefits of exercise and weight loss**		
0–2 (Poor knowledge)	200	92.1
3–4 (Average knowledge)	16	7.4
5–6 (Good knowledge)	1	0.5
**Total**	**217**	**100**
**Score on knowledge about healthy diet**		
0–5 (Poor knowledge)	159	73.3
6–9 (Average knowledge)	58	26.7
10–12 ( Good knowledge)	0	0.0
**Total**	**217**	**100**
**Global knowledge score**		
0–8 (Poor knowledge)	201	92.6
9–13 (Average knowledge)	16	7.4
14–18 (Good knowledge)	0	0.0
**Total**	**217**	**100**

### Attitude towards lifestyle modification

A majority of participants either had a strongly positive attitude (*n* = 112, 51.6%) or a positive attitude (*n* = 71, 32.7%) toward lifestyle modifications – a total of 183 (84.3%) with positive attitudes (Table 3).

**TABLE 3:** Frequency distribution of participants according to attitude score for lifestyle modifications.

Attitude score	Number of respondents	Percentage
0 (Strongly negative)	1	0.5
1 (Negative)	5	2.3
2 (Neutral)	28	12.9
3 (Positive)	71	32.7
4 (Strongly positive)	112	51.6

**Total**	**217**	**100**

### Practice of lifestyle modification

Regarding healthy lifestyle practices, 91.7% (*n* = 199) of the participants reported that they did not exercise regularly; of the 8.3% (*n* = 18) that exercised regularly, the majority (*n* = 17, 94.4%) exercised less than 30 minutes per day or less than 150 minutes per week. Most of the participants (*n* = 217, 99.1%) did not follow a controlled and planned diet and 212 (97.7%) did not monitor their body weight. Table 4 shows the distribution of participants according to their lifestyle modification practice score.

**TABLE 4:** Frequency distribution of participants according to lifestyle modification practice score.

Practice score	Number of respondents	Percentage
0 (Very poor practice)	199	91.7
1 (Poor practice)	13	6.0
2 (Good practice)	3	1.4
3 (Very good practice)	2	0.9

**Total**	**217**	**100**

### Correlation between knowledge, attitude and practice of lifestyle modification

Table 5 shows a positive Pearson correlation of 0.170 (*p* = 0.012) between the global knowledge level and attitude level; a weak, insignificant positive Pearson correlation of 0.037 (*p* = 0.587) between the global knowledge level and practice level; and a weak, positive Pearson correlation of 0.102 (*p* = 0.132) between the attitude level and practice level.

**TABLE 5:** Correlations between global knowledge, attitude and practice level regarding lifestyle modifications (*N* = 217).

Correlation	Pearson correlation	*p*-value
Global knowledge and attitudes	0.17	0.012
Global knowledge and practice	0.04	0.587
Attitudes and practice	0.10	0.132

## Discussion

The majority of participants in this study were in the age groups of 41–50 years (21.6%), 51–60 years (42.9%) and 61–70 years (21.2%). This reflects the fact that type 2 diabetes mellitus usually has its onset after the age of 40 years.^11^ The preponderance of these age groups is also consistent with the findings of the 2003 South African demographic and health surveys in which the majority of South Africans who attended public health facilities in the preceding 30-day period were in the over-45-year age group.^12^

Participants with no formal education (49.5%) and participants with primary school education (43.9%) together constitute an overwhelming 93.4% of the participants in this study. This indicates that most participants have little or no education, a finding which is similar to the results of another South African study which found that 70.6% of black South Africans with type 2 diabetes mellitus had less than a Standard 8 (Grade 10) education.^13^ A majority of the participants were poverty-stricken, with 52.4% earning (ZA) R1000 – R1999, 24.5% earning R500–R999 and 17.9% earning less than R500 per month. Poverty could limit accessibility to and affordability of a well-balanced diet and healthy food and this could explain why the majority of participants had a less-than-acceptable lifestyle modification practice score despite having a positive attitude. This finding is in keeping with the results from a South African income distribution and poverty study where 64% of black South Africans were shown to have a low income.^14^

The high prevalence of overweight (21.6%) and obesity (71%) amongst the participants is similar to the findings in 2009 by another South African study which found that 23% of women were overweight and 41.6% were obese compared with 14.6% and 7.5% of men, respectively, in their study.^15^ Obesity is a major risk factor for type 2 diabetes mellitus and the sedentary lifestyle and lack of physical activity amongst the participants seem to contribute to the high proportion of overweight and obese persons in this study.

The patients’ extremely poor knowledge of lifestyle modification has been replicated in a study in India, in which a majority of respondents (83.3%) had poor knowledge of the advantages of lifestyle modification.^7^ The low level of education amongst the participants, as well as the lack of a well-organised medical nutrition therapy programme within the hospital, may have contributed to this result. In contrast, another study using the same questions found that the majority of respondents in their study (67%) were knowledgeable about lifestyle modifications.^6^ These different finding may be because of the differences in literacy level, training received and availability of information on type 2 diabetes mellitus for their study patients. In this study which used similar questions to assess lifestyle attitude and practices, 47% of their respondents had secondary-level education and had access to a well-organised awareness programme on diabetes mellitus.^6^ This may have accounted for the good lifestyle modification knowledge found in their study participant.

In terms of attitude assessment, the 51.6% of participants with strongly positive attitudes toward lifestyle modification and the 32.7% with positive attitudes jointly constitute a majority 84.3% of the participants with positive attitudes in this study. This is also reflected in other studies, in which the majority of respondents had positive attitudes towards lifestyle modifications.^16, 17^ The proportion of participants with very poor lifestyle practices is similar to a study done in which 75.6% of respondents had bad practices in relation to lifestyle modifications.^8^ This has not been found consistently, as was shown in a study done in India where a majority (99%) of their participants had good lifestyle modification practices.^6^

The positive correlation (*p* = 0.012) between the global knowledge level and the attitude level of participants indicates that the more knowledgeable the participants, the more willing they were to observe lifestyle habits. The very weak and insignificant positive correlation (*p* = 0587) between the global knowledge level and practice level of participants is indicative that being knowledgeable did not necessarily translate into healthy lifestyle practices. In the same vein, the weak insignificant positive correlation (*p* = 0.132) between attitude level and practice level of participants demonstrates that positive attitude did not necessarily translate to good lifestyle modification practices. The correlation trend in this study matches the trend in other studies which found a significant positive correlation (*p* < 0.01) between knowledge and attitude scores.^6^, ^8^ As there is evidence in the literature that good knowledge can be translated into healthy lifestyle practices by promoting behavior change using motivational interview approach,^18, 19^ the lack of knowledge and skills of Mamelodi healthcare workers with regard to motivational behaviour change facilitation might have contributed to this result.

### Limitations of the study

The design of the study did not allow the researchers to determine any causality of the research findings. Secondly, patients at the diabetic clinic of Mamelodi hospital may not be representative of patients with type 2 diabetes mellitus in the general South African population. These limitations notwithstanding, the study revealed important trends that need to be factored into the management of type 2 diabetes mellitus patients, especially in peri-urban black townships.

### Recommendation

A hospital-based lifestyle intervention programme should be implemented in order to improve the knowledge and practice of patients regarding healthy lifestyle. This should be extended to the primary healthcare clinics where the majority of patients are seen.

## Conclusion

The knowledge and practice levels of lifestyle modifications amongst type 2 diabetes mellitus patients attending Mamelodi hospital were generally poor. Nevertheless, majority of these patients have positive attitude toward healthy lifestyle habits which could potentially be harnessed and translated into healthy lifestyle practices.
